# Histological aspects of the small intestine under variable feed restriction: The effects of short and intense restriction on a growing rabbit model

**DOI:** 10.3892/etm.2014.1924

**Published:** 2014-08-21

**Authors:** PETER MAKOVICKY, EVA TUMOVA, ZDENEK VOLEK, PAVOL MAKOVICKY, PAVEL VODICKA

**Affiliations:** 1 Laboratory of Veterinary Histopathology, Komarno, Slovak Republic; 2 Department of Animal Husbandry, Czech University of Life Sciences in Prague, Prague, Czech Republic; 3 Laboratory of Physiology of Nutrition and Quality of Animal Product, Institute of Animal Science, Prague, Czech Republic; 4 Department of Biology, Selye Janos University, Komarno, Slovak Republic; 5 Department of Molecular Biology of Cancer, Institute of Experimental Medicine, Academy of Science of the Czech Republic, Prague, Czech Republic

**Keywords:** histopathology, nutrition, rabbit, small intestine, starvation, intestinal villi

## Abstract

The objective of this study was to investigate the effect of seven days of feed restriction (between days 42 and 49) on the morphology of the small intestine in experimental rabbit models. Sixty weaned Hyplus rabbits (35 days old) were included in the experiment and split into three groups of 20 rabbits. The first control group (n=20) received feed *ad libitum* (ADL group), the second (R1) experimental group (n=20) was fed 50 g feed per rabbit per day and the third (R2) experimental group (n=20) received 65 g feed per rabbit per day. Duodenal samples were collected when the rabbits were aged 49, 56, 63 and 70 days. The mean villus height, crypt depth and small intestine length were measured. Significant interactions (P<0.001) between group and age were identified in the villi height and crypt depths. The maximum mean villus height was found in the R2 group in 56-day-old rabbits (643.14 μm), while the minimum was found in the ADL group in 49-day-old rabbits (460.29 μm). The longest (P<0.001) small intestine was measured in the R1 group in 63-day-old rabbits (347.60 cm), while the shortest was measured in the ADL group in 49-day-old rabbits (263.60 cm). The models show that villus height, crypt depth and the length of the small intestine change with the intensity of feed restriction and age.

## Introduction

The effect of feed restriction has been studied in numerous animal models (1–4), but the question still remains as to what the effects of feed restriction are on health status, the reduction of the risk of separate factors and the utility of the animals. To date, to the best of our knowledge, no experimental study of the effect of different percentages of feed restriction in young rabbit models during their growth phase has been published; thus, the present study was conducted. This study is part of a larger experiment, with other preliminary results published in the Proceedings of the 10th World Rabbit Congress (Sharm El-Sheikh, Egypt) (5–8). There is evidence that the length of starvation induces changes in villus height and crypt depth (9,10). This has already been demonstrated in other animal species, although, to the best of our knowledge, not in young rabbit models.

Rabbit models are very specific. The rabbit is a herbivorous species with functional and morphological specificities of its digestive tract due to the quality of feed ingested. Information on the outcomes of the length and percentage quantity of restriction is important for veterinary practice and clinical medicine, and could be useful for other specific practical purposes. It is necessary to determine the effects of different lengths and intensities of feed restriction, as well as the effect of restriction at different ages, and to verify that there are no pathological effects on the morphology of the small intestine. This can be investigated by observing the changes that occur in intestinal morphology. However, the absorption of nutrients depends on the digestive tract, and it can be assumed that quantitative restriction of feed intake can change the morphology of the small intestine mucosa (11). It well established that the morphology of the intestinal mucosa enables assessment of the dynamics of the differentiation process under normal as well as pathological conditions (12,13). Absorption may be affected by a change in the length of the small intestine and by an entire spectrum of mucosal changes, including the number of villi extending into the lumen. In pathology, measurements of changes in the ratio of villus height to crypt depth are used in documented disorders of the small intestine (14,15). In the present study, we hypothesized that the changes in the length of the small intestine, villus height and crypt depth depend on the restriction time-period and the intensity of feed restriction. The objective of the study was to measure these parameters in growing rabbits under different intensities of feed restriction.

## Materials and methods

This study was approved by the Ethics Committee of the Institute of Animal Science (Prague, Czech Republic) and the Central Commission for Animal Welfare of the Ministry of Agriculture of the Czech Republic (Prague, Czech Republic) and carried out according to the guidelines for applied nutrition experiments in rabbits (16).

### Animals

Sixty Hyplus broiler rabbits (PS 19 × PS 40) were kept in the rabbit building of the Institute of Animal Science under controlled environmental conditions (at a temperature of 16–18°C, with a relative humidity of 65% and 12 h light per day) and housed in cages (50×40×42.5 cm). Three experimental groups (ADL, R1 and R2) were formed. Control rabbits were fed *ad libitum* (ADL group; 174 g/day/animal) during the experiment. Rabbits from the R1 and R2 groups were on feed restriction (50 and 65 g/day/animal, respectively) between 42 and 49 days of age. Animals from both feed restriction groups were fed *ad libitum* prior to and following feed restriction. Rabbits were fed by a commercial type of feed mixture with 165 g crude protein, 183 g crude fiber, 38 g crude fat and 17.1 MJ gross energy/kg (as-fed basis) (Table I). Clinical signs were observed and recorded throughout the experimental period in each individual, specifically clinical signs associated with digestive problems, including diarrhoea, mucus in the faeces and abnormal caecotrophy. The observations were performed in accordance with the methodology of the European Group on Rabbit Nutrition (17).

### Histological studies

For small intestine analysis, five rabbits per group were sacrificed at the ages of 49, 56, 63 and 70 days. Samples of small intestine were obtained by the necropsy method using standard procedures. The entire small intestine from pylorus to ileocaecal junction was first measured. One representative sample was subsequently taken from each animal, 20 cm under the pylorus, and the samples were placed in a 4% Bouin solution (Bamed, České Budějovice, Czech Republic) for histological analysis. All the samples were collected within 30 min after sacrifice. The samples were fixed for one week.

### Sample evaluation

The samples were processed in accordance with standard histological methods. Slices with a 5-μm thickness were cut from each sample and stained with haematoxylin and eosin (Diapath, S.R.L., Martinengo, Italy). Standard slides (Bamed, České Budějovice, Czech Republic) were used for histological purposes. The prepared samples were evaluated under a Nikon Eclipse E600 light microscope (Nikon Corporation Instruments Company, Tokyo, Japan). Villus height and crypt depth were measured using the NIS-Elements version 3.0 software (Nikon Corporation Instruments Company). The height of each villus was measured from the top of the villus to the crypt transition, and the crypt depth was defined as the invagination between two villi. The heights of 40 villi and the depths of 40 crypts were measured per animal.

### Statistical analysis

The data were examined by two-way analysis of variance with group and age interactions using the Statistical Analysis System (SAS) 2003 software (SAS Institute Inc., Cary, NC, USA). P<0.05 was considered to indicate a statistically significant difference.

## Results

### Clinical observations

No clinical signs of digestive problems occurred during the experiment. No significant differences were identified among the groups with regard to live weight. The data collected throughout the experiment are presented in Table II.

### Weight of the rabbits

At the beginning of the experiment the weights of rabbits in the three groups had approximately the same values: ADL, 1,678 g; R1, 1,676 g and R2, 1,751 g. At the end of the experiment the weights of the rabbits had increased: ADL, 2,892 g; R1, 2,650 g and R2, 2,791 g. No significant interaction between group and age was identified.

### Length of the small intestine

It was found that the length of the small intestine significantly (P<0.001) increased with age; however, the level of restriction did not significantly affect this parameter. The longest small intestine was measured in the R1 group in 63-day-old rabbits (347.60 cm) and the shortest in the ADL group in 49-day-old rabbits (263.60 cm).

### Villus height

The data for villus height show interactions between the feeding regime and the age of rabbits. In general, villus height significantly (P<0.001) increased with rabbit age. However, differences were observed between the ADL and restricted rabbits. In the ADL rabbits, the villus height regularly increased with age. In the R1 group, the mean villus height at the age of 49 days, immediately subsequent to restriction, was significantly higher than that in the ADL group. In the following week, the mean villus height in the R1 group was shorter than that in the other two groups. The maximum mean villus height was observed in the R2 group at 56 days; this was significantly different from the heights in the other two groups at the same age.

### Crypt depth

As in the case of villus height, a significant interaction (P<0.001) was detected between age and group for crypt depth. It was found that the ADL group showed a reduction in crypt depth when the depths at the beginning and end of the experiment were compared (from 128.36 to 116.64 μm). Conversely, in the R1 and R2 groups, the crypts were significantly (P<0.001) deeper at 70 days of age in comparison with the depths at the beginning of the experiment (R1, from 123.83 to 132.95 μm; R2, from 116.28 to 145.26 μm).

## Discussion

The physiological functions of the gastrointestinal tract are dependent on the nutrition and quantity of feed. Trying to establish the feed conversion ratio is currently one of the main tasks of all animal breeding sectors. There is a search for the optimal dose of feed that would ensure good yield and also reduce health risk factors.

In rabbits, it is still not known how long a restrictive diet should be applied for and what the effects of restriction for a shorter period of time are on the morphology of the small intestine. Previous studies have shown that starvation is not compatible with a healthy life as it exerts numerous negative effects on the body, although it is common in nature (18,19). The general histological features of the small intestine are well established whereas the histological intestinal alterations induced by ingested feeds are yet to be elucidated (20).

The present study revealed that, following one week of feed restriction in the 49-day-old rabbits, mean villus height was greater than that in the ADL group. However, at the end of the experiment, the mean villus height in the R1 and R2 groups was lower than that in the ADL group. It is possible that, in the absence of nutrients, the villous relief increased, and resulted in higher resorption activity. An increase in resorption may also have been effected by a greater small intestine length. With a gradual adaptation to normal conditions in the realimentation period, the heights of the villi then returned to levels comparable to those of the ADL group. In a previous study, which evaluated the morphometric changes in the small intestine of nursing piglets caused by 60% restriction, it was stated that this restriction resulted in a significantly reduced thickness of the mucosa and villus height compared with the control group (21). In other studies it was revealed that starvation induces proximal small intestine intestinal epithelial atrophy (22,23), which is more closely associated with increased epithelial cell apoptosis (24) than with the full process of cell proliferation in the crypts (25) and is the main cause of increased mucosal permeability (26). In the present study, which was oriented on the histological aspects of feed restriction, no pathological changes were observed. The results show that the small intestine length was shortest in 49-day-old rabbits in the ADL group. The small intestine then increased in length regularly and, at the end of the experiment, was the longest in the ADL group. However, in both restricted groups, the small intestines were longer in the first week of realimentation.

Martignon *et al* (27) stated that a short-term limitation of feed intake appeared not to delay or accelerate the maturation of the small intestine. In the study by Dou *et al* (28), which focused on the structural remodelling in the rat small intestine during starvation, it was stated that the induced morphometric changes in the duodenum were normalised by re-feeding on the second day. These results could explain the compensatory increase in mean villus height in the context of a decrease in the crypt depth. In accordance with the present results, it can be stated that rabbits with functional and morphological specificities of their digestive tract can adapt to starvation in similar ways to other species. This is mainly due to the herbivorous character of their diet and the dependence of the intestines on continuous feed intake. The activity of enterocytes is determined by their enzymatic ability (29,30). It can be suggested that the increased villus height was accompanied by an increasing proliferation of undifferentiated cells and their maturation into full enterocytes. The present results revealed a contrasting trend in the crypt depth to that in the intestinal villi. In the 49-day-old rabbits, the greatest measured crypt depth was in the ADL group. At the end of the experiment, however, the measured value of the crypt depth was higher in the R1 and R2 groups. According to Krizová and Simek (31) the length of the small intestine does not change following intermittent starvation, and animals can adapt to this feed regime. A fundamental investigation demonstrated that the high values of villus height, cell area and cell mitosis were changed by ingested feeds (32). Some of these observations were consistent with those in the present study, with emphasis on the fact that changes in the small intestine are dependent on the species.

## Figures and Tables

**Table I:** Composition of the experimental diet.

Property of feed	Value
Component (%)	
Soybean meal	3.0
Sunflower meal	17.0
Barley	8.0
Oats	9.0
Lucerne meal	30.0
Wheat bran	22.5
Sugarbeet pulp	6.0
Rapeseed oil	1.5
Vitamin supplement	1.0
Ground limestone	1.0
Dicalcium phosphate	0.5
Feeding salt	0.5
Calculated nutrient content (%)	
Crude protein	16.42
Fat	3.86
Crude fibre	18.27
Metabolisable energy (MJ)	9.52

The metabolisable energy applies to 1 kg.

**Table II:** Morphometrical results.

Groups	Age at sampling (days)	Mean VH (μm)	Mean CD (μm)	Mean SIL (cm)	Mean weight (g)
ADL	49	460.29^a^	128.36^b^	263.60	1678
	56	543.98^b^	129.68^b^	312.20	2236
	63	574.03^b,c^	137.18^b,c^	316.60	2525
	70	609.42^b,c^	116.64^b^	340.00	2892
R1	49	517.71^b^	123.83^b^	265.80	1676
	56	511.58^b^	140.52^c^	340.20	2273
	63	545.17^b^	109.97^b^	347.60	2587
	70	606.83^b,c^	132.95^b,c^	336.60	2650
R2	49	461.38^a^	116.28^b^	273.40	1751
	56	643.14^c^	155.56^c^	346.20	2233
	63	553.39^b^	113.08^b,c^	336.40	2428
	70	578.29^b,c^	145.26^c^	331.40	2791
SEM		129.24	46.11	22.17	158.48
Significance (P-value)					
Group		0.069	0.032	0.189	0.111
Age		0.001	0.001	0.001	0.001
Group × age		0.001	0.001	0.598	0.216

Values within a column with different superscripts (^a,b,c^) show statistically significant differences (P<0.05). Differences express interaction of age and group. VH, villus height; CD, crypt depth; SIL, small intestine length; ADL, *ad libitum*; R1, restriction of 50 g food per rabbit per day; R2, restriction of 65 g food per rabbit per day; SEM, standard error of the mean.
